# Supplementary material for: Exposure to airborne bacteria depends upon vertical stratification and vegetation complexity
Source: Sci Rep. 2021 May 4;11:9516. doi: 10.1038/s41598-021-89065-y (PMC8096821; doi:10.1038/s41598-021-89065-y)
Supplement: Supplementary file 2 — Supplementary Information 2. [file 41598_2021_89065_MOESM2_ESM.docx]

**Supplementary Materials**

Exposure to Airborne Bacteria Depends upon Vertical Stratification and Vegetation Complexity

**Jake M. Robinson^1,2,3,4*^, Christian Cando-Dumancela^3,4^, Rachael E. Antwis^5^, Ross Cameron^1^, Craig Liddicoat^3,4,6^, Ravin Poudel^7^, Philip Weinstein^4,6^, and Martin F. Breed^3,4^**

***Appendix A***

A minimum of 0.20 ng/uL of usable PCR product was required in order to generate sequencing output guarantee of 10,000 raw reads and to be included in the analysis.

Using PEAR (version 0.9.5), paired-end reads were assembled by aligning the forward and reverse reads. We then identified and trimmed the primers. To process the trimmed reads, we used Quantitative Insights into Microbial Ecology (QIIME1.8), USEARCH (version 8.0.1623), and UPARSE software. Reads were quality filtered, and we removed full-length duplicate reads and sorted them by abundance using USEARCH tools. We discarded singletons or unique reads in the data set. We clustered the reads, and chimeric reads were filtered using the “rdp_gold” database as a reference. To obtain the number of reads in each operational taxonomic unit (OTU), we mapped the reads back to OTUs with a minimum identity of 97%. We assigned taxonomy using QIIME1.8.

We used the phyloseq package in R to import and analyse the sequencing data, and decontam to identify and exclude contaminants.

Lower biomass samples (i.e., air, field blanks, and extraction blank controls) were analysed using the isNotContaminant() function, where contaminants were identified by increased prevalence in negative controls. Higher biomass samples (i.e., soil, and corresponding extraction blanks) were analysed using the isContaminant() function. Using isContaminant(), contaminants were identified by the frequency that varies inversely with sample DNA concentration, or by increased prevalence in negative controls. All taxa identified as contaminants were pooled and removed from further analysis.

***Appendix B***

In the samples from the scrub habitat, we obtained 3,781,284 raw sequencing reads from air samples with an average length of 300 bp, with 3,278,433 reads kept after quality control (QC). For the soil samples, we obtained 1,830,395 raw reads and 1,287,303 reads after QC. The range of reads per sample after QC was 19,966-251,822. Reads were clustered into 10,563 OTUs, with 157 OTUs unclassified at the phylum level, and 6003 at the genus level. In bare ground samples, we obtained 1,958,593 raw reads from air samples with an average length of 300 bp and 1,344,612 reads after QC. For the soil samples, we obtained 486,431 raw reads and 397,443 reads after QC. The range of reads per sample after QC was 24,971-220,055. Reads were clustered into 6,755 OTUs, with 50 OTUs unclassified at the phylum level, and 5017 at the genus level. In grassland samples, we obtained 1,770,620 raw reads from air samples with an average length of 300 bp and 1,405,350 reads after QC. For the soil samples, we obtained 547,496 raw reads and 412,556 reads after QC. The range of reads per sample after QC was 23,338-166,874. Reads were clustered into 6,420 OTUs, with 90 OTUs unclassified at the phylum level, and 4527 at the genus level.


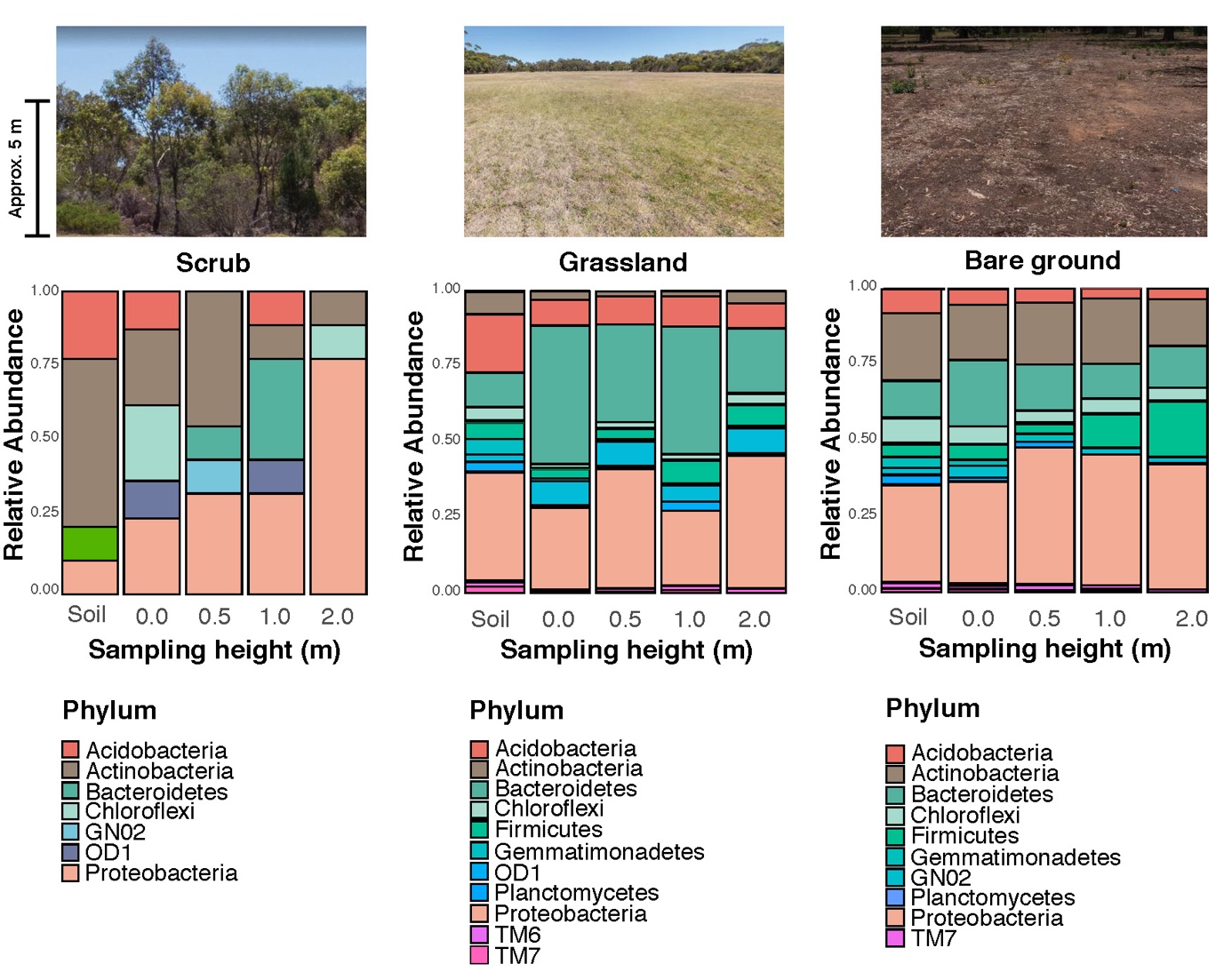


**Figure S1.** Profile of bacterial communities from each habitat at the phylum level. The coloured area of each bar represents the relative abundance of the corresponding phylum over 1%. The X-axis displays sampling heights: soil, 0.0 m, 0.5 m, 1.0 m, and 2.0 m (from left to right). The photographs above the plots show examples of each habitat used in the study (photographs by authors).

***Appendix C***

**Table S1.** Shannon alpha diversity values with mean and standard deviation for each habitat type. These are further divided into all samples, air only, and soil only showing values for each Day (1, 2 and 3) and for each Site (1, 2 and 3).

| **Habitat type ↓** | **Day 1 mean Shannon α diversity value (±**  **SD)** | **Day 2 mean Shannon α diversity value (±**  **SD)** | **Day 3 mean Shannon α diversity value (±**  **SD)** | **Site 1 mean Shannon α diversity value (±**  **SD)** | **Site 2 mean Shannon α diversity value (±**  **SD)** | **Site 3 mean Shannon α diversity**  **value (±**  **SD)** |
| --- | --- | --- | --- | --- | --- | --- |
| Bare ground all samples | 3.58 (1.73) | 3.89 (1.85) | 3.90 (1.44) | 4.50 (1.25) | 3.47 (1.54) | 3.36 (1.75) |
| Bare ground air only | 3.01 (1.49) | 3.41 (1.64) | 3.41 (1.31) | 4.25 (1.29) | 2.86 (1.33) | 2.74 (1.28) |
| Bare ground soil only | 5.69 (0.25) | 5.62 (0.33) | 5.56 (0.27) | 5.55 (0.24) | 5.70 (0.00) | 5.61 (0.16) |
| Grassland all samples | 3.35 (1.42) | 3.45 (1.64) | 3.28 (1.54) | 3.64 (1.63) | 2.90 (1.46) | 3.54 (1.41) |
| Grassland air only | 2.83 (1.04) | 2.97 (1.46) | 2.70 (1.11) | 3.11 (1.36) | 2.33 (0.96) | 3.07 (1.14) |
| Grassland soil only | 5.44 (0.24) | 5.37 (0.32) | 5.57 (0.33) | 5.75 (0.10) | 5.20 (0.02) | 5.43 (0.26) |
| Scrub all samples | 4.71 (1.21) | 4.73 (1.10) | 4.74 (0.82) | 4.56 (1.18) | 4.77 (0.97) | 4.90 (0.98) |
| Scrub air only | 4.43 (1.22) | 4.46 (1.09) | 4.41 (0.59) | 4.28 (1.16) | 4.55 (0.96) | 4.49 (0.81) |
| Scrub soil only | 5.76 (0.16) | 5.73 (0.18) | 5.85 (0.21) | 5.69 (0.05) | 5.66 (0.08) | 5.85 (0.21) |

**Table S2.** PERMANOVA results for assessment of inter-date and inter-site variation in bacterial community composition.

|  | **Df** | **MeanSqs** | **F.Model** | **R2** | **P-value** |
| --- | --- | --- | --- | --- | --- |
| **Inter-date** | | | | | |
| Scrub | 2 | 6056 | 0.7 | 0.03 | 0.82 |
| Bare ground | 2 | 3964 | 0.9 | 0.03 | 0.54 |
| Grassland | 2 | 3829 | 1.0 | 0.04 | 0.41 |
| **Inter-site** | | | | | |
| Scrub | 2 | 1075 | 1.3 | 0.05 | 0.11 |
| Bare ground | 2 | 4382 | 1.0 | 0.06 | 0.36 |
| Grassland | 2 | 5484 | 1.3 | 0.05 | 0.40 |

**Table S3.** Statistical comparison of central tendency between sites and dates for each habitat type (using Chi-squared or ANOVA tests).

| **Habitat type ↓** | **Inter-date air and soil** | **Inter-date air only** | **Inter-date soil only** | **Inter-site air and soil** | **Inter-site air only** | **Inter-site soil only** |
| --- | --- | --- | --- | --- | --- | --- |
| Bare ground | Chi-squared=0.34, df=2, *P*=0.84 | Chi-squared=0.67, df=2, *P*=0.71 | ANOVA F=0.36, df=1, *P*=0.57 | Chi-squared=0.33, df=2, *P*=0.84 | Chi-squared=7.27, df=2, *P*=0.03 | ANOVA F=0.07, df=1, *P*=0.79 |
| Grassland | ANOVA F=0.03, df=1, *P*=0.86 | ANOVA F=0.07, df=1, *P*=0.79 | ANOVA F=0.29, df=1, *P*=0.60 | ANOVA F=0.19, df=1, *P*=0.89 | ANOVA F=0.006, df=1, *P*=0.94 | ANOVA F=2.3, df=1, *P*=0.17 |
| Scrub | Chi-squared=0.04, df=2, *P*=0.98 | ANOVA F=0.001, df=1, *P*=0.97 | ANOVA F=0.38, df=1, *P*=0.55 | Chi-squared=0.46, df=2, *P*=0.79 | ANOVA F=0.27, df=1, *P*=0.60 | ANOVA F=9.1, df=1, *P*=0.02 |

**
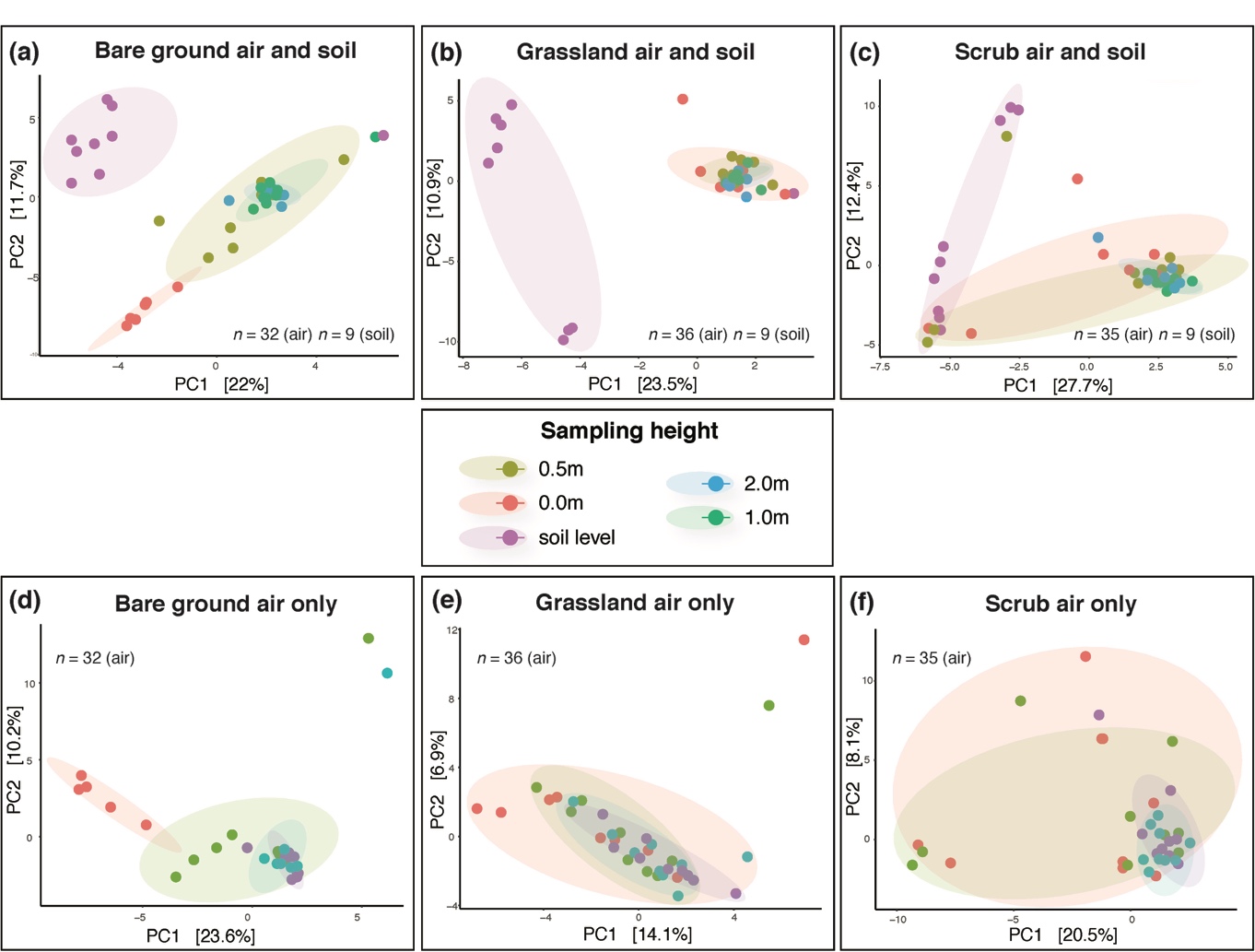
**

**Figure S2.** Visualising bacterial beta di­versity with ordination plots of Aitchison distances based on clr-transformations of OTU abundances for each sampling height across the three habitats: (a) Bare ground air and soil, (b) Grassland air and soil, (c) Scrub air and soil, (d) Bare ground air only, (e) Grassland air only, and (f) Scrub air only. Ellipses represent Euclidian distance from the centre – with the radius equal to the confidence level (0.95). Clusters suggest differences between communities at different sampling heights (indicated by the colours).

Tables S4-S6 show Pearson’s correlations testing for vertically stratified bacterial alpha diversity in the bare ground, grassland, and scrub habitats. These results include a breakdown of correlations across dates and sites.

**Table S4.** Pearson’s correlations results for vertically stratified bacterial alpha diversity in the bare ground habitat.

| **Days/sites** | ***r* score** | ***df*** | ***P-value*** |
| --- | --- | --- | --- |
| Day 1 (04-11-19) | -0.83 | 12 | <0.01*** |
| Day 2 (05-11-19) | -0.70 | 12 | <0.01*** |
| Day 3 (06-11-19) | -0.74 | 11 | <0.01*** |
| Bare ground 01 (B01) | -0.76 | 11 | <0.01*** |
| Bare ground 02 (BG02) | -0.84 | 13 | <0.01*** |
| Bare ground 03 (BG03) | -0.84 | 11 | <0.01*** |

**Table S5.** Pearson’s correlations results for vertically stratified bacterial alpha diversity in the Grassland habitat.

| **Days/sites** | ***r* score** | ***df*** | ***P-value*** |
| --- | --- | --- | --- |
| Day 1 (04-11-19) | -0.54 | 13 | 0.03* |
| Day 2 (05-11-19) | -0.26 | 13 | 0.35 |
| Day 3 (06-11-19) | -0.36 | 13 | 0.19 |
| Grassland 01 (GR01) | -0.49 | 13 | 0.06 |
| Grassland 02 (GR02) | -0.49 | 13 | 0.06 |
| Grassland 03 (GR03) | -0.33 | 13 | 0.23 |

**Table S6.** Pearson’s correlations results for vertically stratified bacterial alpha diversity in the Scrub habitat.

| **Days/sites** | ***r* score** | ***df*** | ***P-value*** |
| --- | --- | --- | --- |
| Day 1 (04-11-19) | -0.76 | 12 | <0.01*** |
| Day 2 (05-11-19) | -0.38 | 12 | 0.17 |
| Day 3 (06-11-19) | -0.65 | 11 | 0.01** |
| Scrub 01 (SC01) | -0.69 | 13 | <0.01*** |
| Scrub 02 (SC02) | -0.43 | 13 | 0.11 |
| Scrub 03 (SC03) | -0.73 | 9 | 0.01** |

***Environmental metadata***

We ran Spearman’s correlations with bootstrap resampling to determine whether environmental metadata (pH, temperature, windspeed) associated with bacterial alpha diversity (Table S6). There were no significant associations between the metadata and bacterial alpha diversity except for windspeed and aerobiome alpha diversity (*rs* = -0.57, *ß* = 0.57 (-0.78 – -0.22), *P* = 0.01). Analysis of individual habitats showed that the significant association only remained for scrub habitat, with aerobiome alpha diversity decreasing significantly as windspeed increased (*rs* = -0.88, *ß* = -0.88 (-0.98 – -0.5), *P* = <0.01). Average windspeed was significantly different between habitats (ANOVA F = 27, df = 2, *P* = <0.01). Windspeed was lower in scrub (x̅ = 1.16 m/s) compared to grassland (x̅ = 4.45 m/s) and bare ground (x̅ = 3.29 m/s), confirmed by a Tukey multiple comparison of means test (*P* = <0.05).

**Table S7.** Spearman’s correlations and bootstrap sampling results for metadata and bacterial alpha diversity.

| **Test** | **rs** | **P-value** | **ß (2.5% and 97.5% CI)** |
| --- | --- | --- | --- |
| Alpha air ~ pH | 0.09 | 0.65 | 0.09 (-0.33 – 0.49) |
| Alpha soil ~ pH | 0.05 | 0.81 | 0.05 (-0.36 – 0.43) |
| Alpha air ~ temperature | -0.28 | 0.14 | -0.29 (-0.64 – 0.15) |
| Alpha soil ~ temperature | 0.06 | 0.75 | 0.06 (-0.36 – 0.46) |
| Alpha air ~ windspeed | -0.58 | <0.01 | -0.57 (-0.87 - -0.22) |
| Alpha soil ~ windspeed | -0.19 | 0.34 | -0.19 (-0.53 – 0.19) |

***Appendix D***


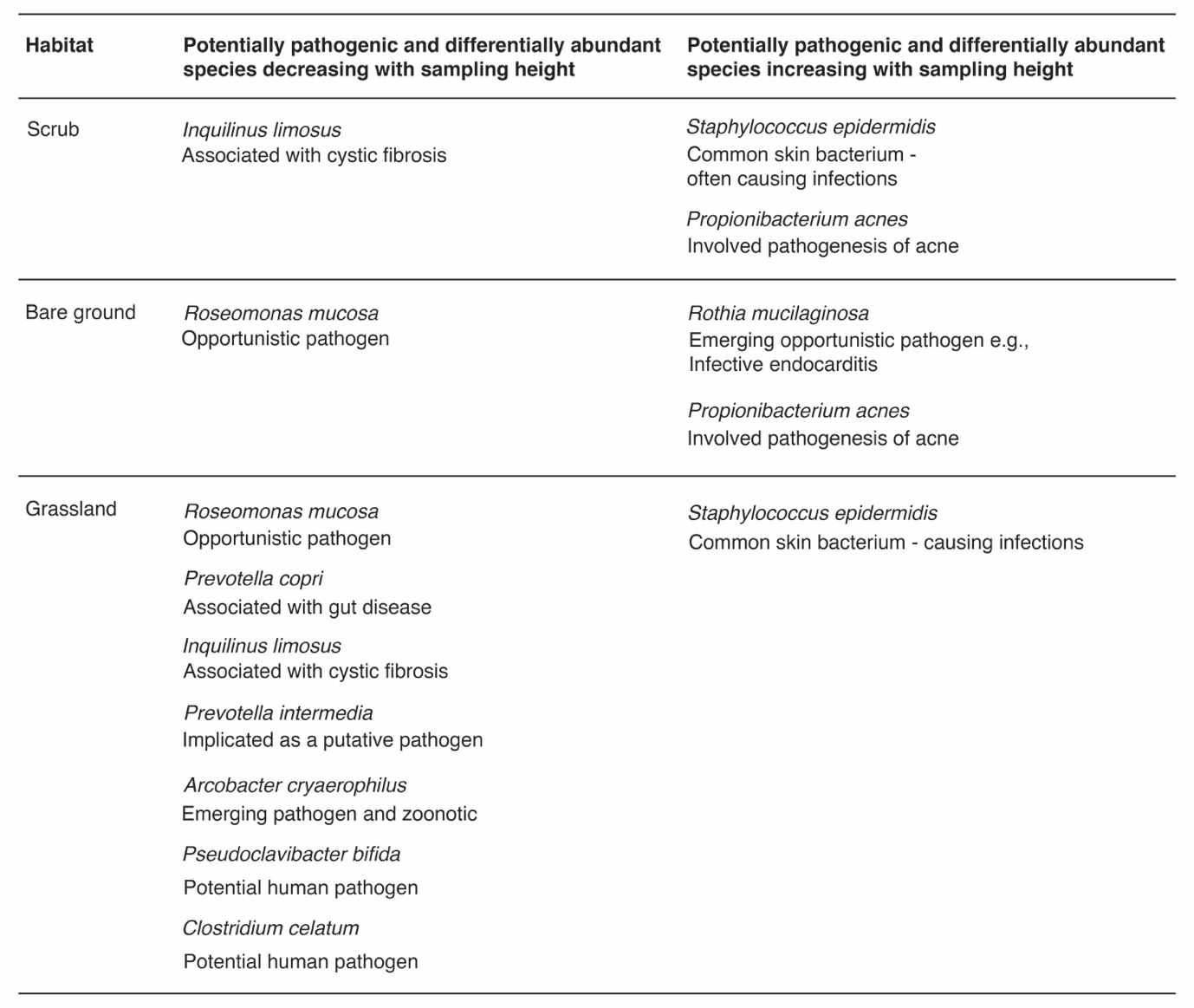


**Figure S3**. List of identified potential human pathogens and their associated diseases
